# Independent component analysis for internet gaming disorder

**DOI:** 10.1080/19585969.2023.2168135

**Published:** 2023-02-07

**Authors:** Gemma Mestre-Bach, Roser Granero, Fernando Fernández-Aranda, Susana Jiménez-Murcia, Marc N. Potenza

**Affiliations:** a Facultad de Ciencias de la Salud, Universidad Internacional de la Rioja, La Rioja, Spain; b Institute for Culture and Society (ICS), University of Navarra, Pamplona, Spain;; c Departament de Psicobiologia i Metodologia de les Ciències de la Salut, Universitat Autònoma de Barcelona, Barcelona, Spain; d Ciber Fisiopatología Obesidad y Nutrición (CIBERObn), Instituto de Salud Carlos III, Madrid, Spain; e Psychoneurobiology of Eating and Addictive Behaviors Group, Neurosciences Programme, Bellvitge Biomedical Research Institute (IDIBELL), Barcelona, Spain; f Behavioral Addictions Unit, Department of Psychiatry, Bellvitge University Hospital, Barcelona, Spain; g Department of Clinical Sciences, School of Medicine and Health Sciences, University of Barcelona, Barcelona, Spain; h Department of Psychiatry, Yale University School of Medicine, New Haven, CT, USA; i Department of Neuroscience, Yale University School of Medicine, New Haven, CT, USA; j Child Study Center, Yale University School of Medicine, New Haven, CT, USA; k Connecticut Council on Problem Gambling, Wethersfield, CT, USA; l Connecticut Mental Health Center, New Haven, CT, USA; m Wu Tsai Institute, Yale University School of Medicine, New Haven, CT, USA

**Keywords:** Internet gaming disorder, independent component analysis, fMRI, default-mode network, executive-control network, salience network

## Abstract

**Introduction:** There is a growing interest in the study of the neurobiological correlates of internet gaming disorder (IGD), and new techniques are beginning to be implemented for this purpose, such as independent component analysis (ICA).

**Aims:** The present narrative review aimed to explore the studies that had used ICA for the study of the different brain networks possibly associated with IGD.

**Methods:** We specifically focussed on three of the main networks: default-mode network, executive-control and salience networks.

**Results:** Most studies have identified alterations in these three brain networks in individuals with IGD, which may be involved in the development and maintenance of this disorder.

**Conclusion:** More studies are needed to deepen an understanding of the specific role of each in the symptomatology and treatment of IGD.

## Introduction

Internet Gaming Disorder (IGD) was recommended as a mental disorder in Section III of the Diagnostic and Statistical Manual of Mental Disorders (DSM-5) (American Psychiatric Association 2013), and gaming disorder has been recognised in the 11th revision of the International Classification of Disease (ICD-11) (World Health Organization 2020) as a ‘disorder due to addictive behaviors’. IGD has been understood as a persistent and recurrent pattern of internet gaming involvement despite physical/psychological impairment.

The Interaction of Person-Affect-Cognition-Execution (I-PACE) model has proposed that IGD, as well as other addictive behaviours, is the consequence of interactions that occur between core characteristics of an individual and multiple moderating and mediating variables (Brand et al. 2016, 2019). At a neurobiological level, this model suggests that the development and maintenance of IGD are associated with an imbalance between (a) an increase in incentive-oriented urges and desires; and (b) a reduction in situation-specific inhibitory control over these urges and desires (Brand et al. 2019). Therefore, individuals with reward deficiencies are more likely to develop incentive sensitisation (as a result of conditioning processes), which may be associated with attentional biases, cue-reactivity and craving, factors that may contribute to engagement in IGD and other addictions (Brand et al. 2019).

The neural correlates of IGD involved in this imbalance have been explored in different functional magnetic resonance imaging (fMRI) studies, and some have used independent component analysis (ICA). The exploration of neural correlates or biomarkers (understood as any structure, process, or substance that can be measured objectively) is important in IGD due to the increasing prevalence of, and morbidity related to, IGD (Strimbu and Tavel 2010; Kashif et al. 2021). The identification of biomarkers could facilitate improved understanding that could lead to better prevention and treatment strategies for IGD (Kashif et al. 2021). In the present review, the main findings of these studies using ICA will be discussed (Table 1), taking into account three main brain connectivity networks: the default-mode network (DMN), the executive-control network (ECN) and the salience network (SN).

**Table 1.** Main studies using ICA to explore the neurobiology of IGD.

Reference	Country	Aims	Sample	IGD assessment	Task	Results
Lee et al. (2020)	South Korea	To investigate the functional connectivity between large-scale intrinsic networks including the default-mode, executive-control and salience networks.	17 male adolescents with IGD; 18 male HCs	Korean Internet Addiction Proneness scale; 9 DSM-5 criteria for IGD	Resting state	Differential FC of the SN and DMN with the left pSTS was observed in male adolescents with IGD. FC between the SN and pSTS correlated with severity of internet addiction and self-reported cognitive problems. ICA revealed that pSTS was involved in a social brain network.
Ma et al. (2019)	China	To investigate the activity of temporally coherent, large-scale FNs during cue-reactivity in IGD.	29 male individuals with IGD; 23 male HCs	Scores of the CIAS ≥67; Spent more than half of the time online on games; Spent ≥14 h on internet gaming per week (i.e., with averagely at least 2 h spent on internet gaming every day)	Cue-reactivity task involving internet-gaming stimuli (i.e., game cues) and general internet surfing-related stimuli (i.e., control cues)	4 FNs were identified that were related to the response to game cues relative to control cues and that showed altered engagement/disengagement in IGD in comparison with HCs. These FNs included temporo-occipital and temporo-insula networks (related to sensory processing), a frontoparietal network (involved in memory and executive functioning) and a dorsal-limbic network (involved in reward and motivation processing). Within IGD participants, game versus control engagement of the temporo-occipital and frontoparietal networks were positively linked with IGD severity. Similarly, disengagement of the temporo-insula network was negatively correlated with higher game-craving.
Ma et al. (2021)	China	(1) To examine fMRI data of the stop-signal task and compare individuals with IGD and HCs in neural responses during post-error behavioural adjustment. (2) To investigate the neural representation of probabilistic expectations of the stop signal during the stop-signal task.	21 male young adults with IGD; 21 male HCs	Meeting at least 5 of the 9 DSM-5 criteria for IGD; Scores ≥50 on the IAT; Engagement in internet gaming for >20 h per week for a minimum of 1 year; internet gaming as the primary online activity	Stop-signal task	Diminished engagement of the fronto-parietal network during post-error slowing was observed, as well as weaker activity in the ventral attention and anterior DMN in response to stop signals in IGD compared to HCs.
Wang et al. (2016)	China	To identify the neural mechanism of risky decision-making in IGD using a probability discounting task.	19 male individuals with IGD; 21 male HCs	IAT; 9 DSM-5 criteria for IGD	Probability discounting task	The individuals with IGD showed greater task-related activity in DMN and less engagement in the ECN in comparison with HCs during risky decisions. The engagement of the DMN was negatively associated with the reaction time and the ECN engagement was positively linked with the probability discounting rates.
Wang et al. (2017)	China	To explore FC in male participants with IGD.	18 male individuals with IGD; 21 male HCs	IAT	Delay-discounting task	Two networks were linked with IGD: (a) the ECN containing the anterior cingulate cortex and the medial and superior frontal gyrus; and, (b) the basal ganglia network containing the lentiform nucleus. Individuals with IGD showed stronger FC when selecting small and now options, in comparison with HCs. In addition, the delay-discounting rates were positively associated with the modulation of the 2 networks and reaction times. The results suggest that individuals with IGD may have enhanced sensitivity to reward and decreased ability to control their impulsivity effectively, which may lead to myopic decision-making.
Wang, Zhang et al. (2018)	China	To explore alterations in related functional brain networks underlying attentional biases in individuals with IGD.	18 male individuals with IGD; 19 male HCs	IAT; 9 DSM-5 criteria for IGD	Addiction Stroop task	ICA identified 4 functional networks that showed differences between both groups, which were related to the right ECN and visual networks. Right ECN: individuals with IGD showed increased FC in the temporal gyrus and frontal gyrus, and reduced FC in the posterior cingulate cortex, temporal gyrus and frontal gyrus, in comparison with HCs.
Wang et al. (2019)	China	To explore differences between the engagement of neuronal networks of individuals with IGD and that of RGUs.	18 male individuals with IGD; 20 male RGUs	IAT; 9 DSM-5 criteria for IGD	Probability discounting task	ICA analysis showed that individuals with IGD have stronger FC in reward circuits and their ECN, as well as lower FC in the ASN than RGUs.
Xing et al. (2014)	China	To investigate whether the structural connectivity and FC within SN were altered in adolescents with IGD, and to assess impaired cognitive control in adolescents with IGD.	17 adolescents with IGD; 17 adolescent HCs	IAT; LOL as their primary mainly Internet activity	Stroop colour-word task	Impaired cognitive control in IGD was suggested by more errors during the incongruent condition in the colour-word Stroop task. The right SN tract showed the decreased FA in adolescents with IGD, though no significant differences in FC were detected. The FA values of the right SN tract were negatively correlated with the errors during the incongruent condition in IGD adolescents.
Yip et al. (2018)	China	To compare neural responses between drug-naïve young adults with and without IGD during performance of an emotion-regulation task using both whole-brain GLM and ICA approaches.	28 male individuals with IGD; 28 male HCs	9 DSM-5 criteria for IGD	Emotio-regulation task	Individuals with IGD showed significantly blunted neural responses within distributed subcortical and cortical regions including the striatum, insula, lateral prefrontal cortex and anterior cingulate in response to negative affective cues, as well as during emotion regulation, in comparison with HCs. ICA further identified between-group differences in engagement of a fronto-cingulo-parietal network, involving decreased engagement in individuals with IGD, compared to HCs.
Yuan et al. (2016)	China	To investigate the abnormal brain network interactions using multimodal imaging between adolescents with IGD and HCs.	28 adolescents with IGD; 25 adolescent HCs	YDQ; LOL as their primary mainly internet activity	Stroop colour-word task	Abnormal FC within CENs and effective connectivity within the SN in IGD adolescents were detected, and inefficient interactions between both brain networks were observed. Reduced fractional anisotropy in the SN and right CEN tracts, and between-network (the anterior cingulate cortex-right dorsolateral prefrontal cortex tracts) pathways were identified in individuals with IGD. A significant correlation between the effective and structural connection from SN to CENs and the number of errors during the incongruent condition in the Stroop task were identified in individuals with IGD and HCs.
Zeng et al. (2022)	China	To investigate the effective connectivity patterns differences of frontostriatal circuits between individuals with IGD and HCs.	148 individuals with IGD; 169 RGUs	IAT; 9 DSM-5 diagnostic criteria for IGD	Resting-state fMRI data	Individuals with IGD showed inhibitory effective connectivity from the right OFC to the right caudate and from the right dorsolateral prefrontal cortex to the left OFC, compared with RGUs. Excitatory effective connectivity was observed from the thalamus to the left OFC. Correlation analyses showed that the directional connection from the right OFC to the right caudate was negatively linked with addiction severity.
Zhang et al. (2017)	China	To assess alterations in the inter-network interactions of large-scale networks in IGD, and to associate the alterations with IGD-related behaviours.	39 males with IGD; 34 male HCs	Score ≥67 of the CIAS; Spent more than half of the online time on internet games; 14 h or more spent on internet gaming per week (with at least 2 h spent on internet gaming every day)	Resting-state fMRI data	Individuals with IGD had significantly increased SN-DMN connectivity and decreased RAI connectivity in the right hemisphere (rRAI), compared with HCs. The rRAI measures in IGD participants were negatively associated with their craving scores.
Zeng et al. (2022)	China	ICA was used to determine the relevant components of the brain to make comparisons among IGD, TUD and HC groups through intra-network analysis and inter-network analysis.	92 individuals with IGD; 96 individuals with TUD\; 107 HCs	DSM-5 criteria for IGD; Scores more than 50 in IAT	Resting-state fMRI data	Significant differences in the subcortical network and cerebellar network were found using ICA.

Abbreviations. ASN: anterior salience network; CENs: central executive networks; CIAS: Chinese Internet Addiction Scale; DMN: default mode network; DSM: Diagnostic and Statistical Manual of Mental Disorders; ECN: executive-control network; FA: fractional anisotropy; FC: functional connectivity; fMRI: functional magnetic resonance imaging; FNs: functional brain networks; GLM: general-linear-model; HCs: healthy controls; IAT: Internet addiction test; IC: independent component; ICA: independent component analysis; IGD: Internet gaming disorder; LOL: League of Legends; MELODIC: Multivariate Exploratory Linear Decomposition into Independent Components; mPFC: medial prefrontal cortex; OFC: orbitofrontal cortex; pSTS: posterior superior temporal sulcus; RAI: resource allocation index; RGU: recreational Internet game users; TUDs: tobacco use disorders; YDQ: Young Diagnostic Questionnaire for Internet addiction criteria.

## Independent component analysis in internet gaming disorder

Data-driven methodologies have advantages over some other approaches in that they may facilitate the identification of novel, unanticipated findings and thus propel fields of research. When applied to neuroimaging data, ICA is a blind-source separation technique that may allow the identification of distinct groups of brain regions that exhibit the same temporal pattern of homodynamic signal change. Furthermore, it does not rely on any experimental design matrix or any other prior information about the temporal response (Wang, Zhang et al. 2018). Therefore, it is a method that belongs to blind signal-separation methods that assume that there is statistical independence of the source signals, and which allows the isolation of these different sources (McKeown et al. 2003). In order to identify these source signals, covariance measures are necessary and higher-order statistic algorithms (such as FastICA, Infomax, or JADE) or intra-source correlation methods (such as Molgedey and Schuster, and Ziehe and Muller) are often used (McKeown et al. 2003).

Some authors support its usefulness given the limitations of general linear modelling (GLM), a widely used index to explore task-specific brain responses. This is done by analysing a time series of blood-oxygen-level dependent signals. However, this index requires an experimental design matrix and does not allow the identification of a specific brain region that is functionally connected to another (Wang, Zhang et al. 2018). The same applies to the more classical methods of factor analysis and principal component analysis, which, as ICA, can decompose the data, but may not find underlying sources (Hyvärinen 2013).

In recent years, ICA has begun to be more widely used in the mental health field, in disorders such as schizophrenia (Brandt et al. 2015; Gupta et al. 2017), depression (Maglanoc et al. 2020; Liu, Jiao, et al. 2021; Luo et al. 2022), anxiety (Ni et al. 2021) and bipolar (Yip et al. 2014; Tang et al. 2020) disorders. In the addictions field, it has been used in the study of both substance-use disorders and addictive behaviours, and mostly in cocaine-use disorder (Zhang et al. 2016; Wang, Worhunsky et al. 2018; Worhunsky et al. 2021), and IGD (Bin et al. 2022; Zeng et al. 2022). The present narrative review aims to examine the main studies that have used ICA in individuals with IGD in order to gain insight into the neurobiology of this mental disorder.

### Default-mode network

**Figure 1.**
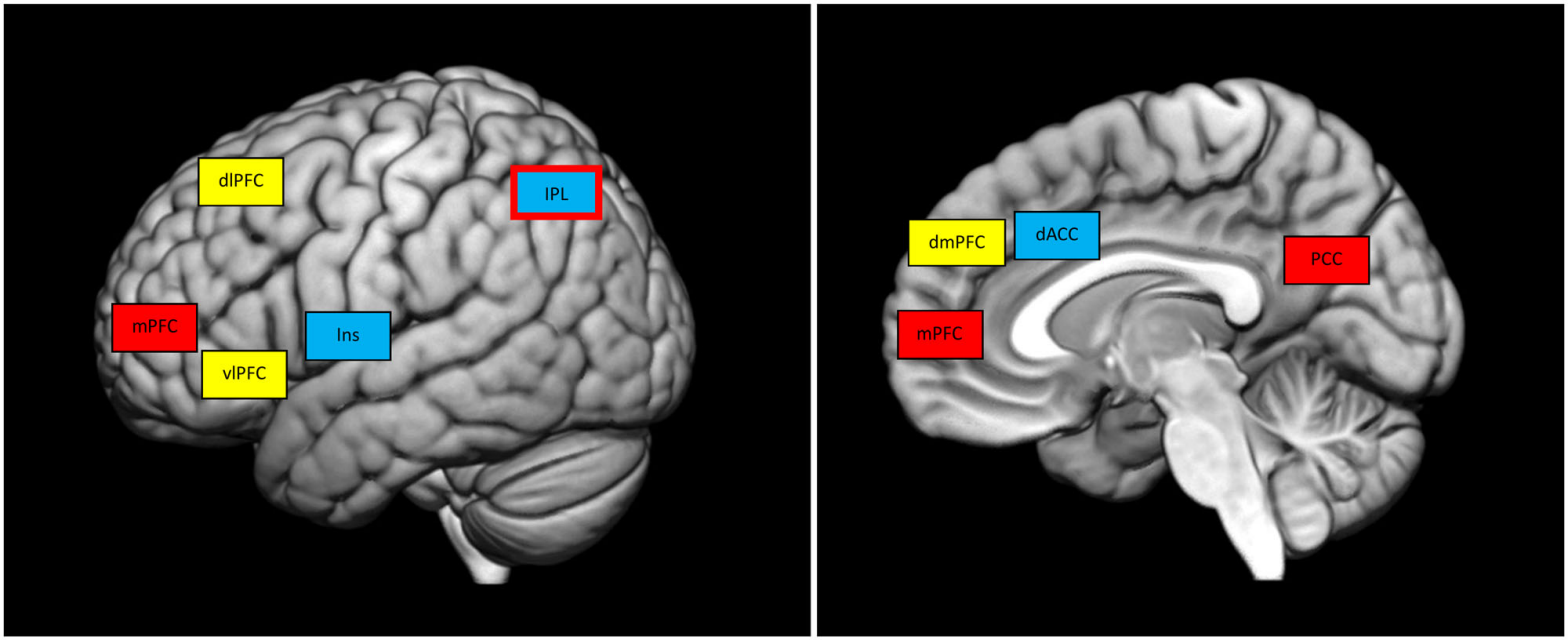
Main brain areas of the different networks. 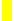 Executive-control network. dlPFC: dorsolateral prefrontal cortex; vlPFC: ventrolateral prefrontal cortex; dmPFC: dorsomedial prefrontal cortex. 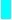
Salience network. IPL: inferior parietal lobe; dACC: dorsal anterior cingulate cortex; Ins: insula. 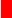
Default mode network. IPL: inferior parietal lobe; mPFC: medial prefrontal cortex; PCC: posterior cingulate cortex.

The brain’s DMN encompasses different networks present in different areas of the association cortex (Buckner and DiNicola 2019). Specifically, the main brain regions involved in the DMN are the posterior cingulate cortex (PCC), the medial prefrontal cortex (MPFC) and the inferior parietal lobule (IPL) (Di and Biswal 2014). The DMN has been implicated in high-level cognitive processes, such as emotional-regulation processes, autobiographical memory and self-related cognition, social cognition, risky decision-making, impulsivity and future-oriented thinking (Buckner and DiNicola 2019; Yan et al. 2021; Figure 1).

In the case of IGD, individuals may show less connectivity with DMN brain areas (Liu, Potenza, et al. 2021), and an altered functional interaction between the DMN and the ventral-attentional network. This may increase incentive salience, that is, attentional bias, and reduce cognitive control (Yan et al. 2021). Although the specific role of DMN in the development and maintenance of IGD remains unclear (Yan et al. 2021), it has been suggested that less connectivity in the DMN may be a neural marker of the disorder, which contributes importantly to the differentiation between individuals with IGD and recreational game use (Dong et al. 2020), as well as between IGD and non-gaming control participants (Dong et al. 2017). Additionally, although IGD was not included in this study, a craving network identified across conditions suggests that craving may involve difficulties redirecting attention from internal self-related processes as linked to the DMN (Garrison et al. 2021). Given the relevance of craving to IGD, additional research should explore this possible role for the DMN in IGD. Some authors have suggested that the DMN may be the most informative network in predicting the presence of IGD (Song et al. 2021).

Some studies have explored a specific role for the DMN in IGD using ICA. Lee et al. (2020) used ICA to explore the DMN (focusing on the PCC) in 17 male adolescents with IGD and 18 control participants. The authors found aberrant functional connectivity of DMN (specifically involving the PCC) with the left posterior superior temporal sulcus (pSTS) only in the group of adolescents with IGD. The DMN, specifically the anterior DMN (aDMN), was also explored through ICA by Ma et al. (2021) in a sample of 21 male young adults with IGD and 21 control participants. The authors observed that the aDMN showed greater disengagement during stop-signal anticipation. This could reflect a lower attentional and monitoring processing of unexpected cues and, consequently, a suboptimal disposition to withhold response in the case of individuals with IGD. Wang et al. (2016) through ICA, observed that 19 individuals with IGD included in their study showed higher task-related activity in DMN, compared to control participants.

### Executive-control network

The ECN involves fronto-parietal regions comprising the dorsolateral prefrontal cortex (dlPFC), the ventrolateral prefrontal cortex (VLPFC) and the posterior parietal cortex (PPC). This network has been implicated in top-down cognitive-control functions, such as the maintenance and manipulation of information in working memory, as well as the cognitive control of thoughts, emotions and behaviours, in rule-based problem-solving and decision-making in goal-directed behaviours (Menon 2011; Cole et al. 2013; Figure 1).

Some studies have explored specific roles for the ECN in IGD through ICA. Ma et al. (2019) explored, through ICA, the activity of temporally coherent, large-scale functional networks during cue reactivity in a sample of 29 male individuals with IGD and 23 control participants. The authors examined, among others, the ECN (especially dlPFC, middle and inferior frontal cortex, middle temporal gyrus and inferior parietal lobe), highlighting an association between ECN and IGD severity (mainly hours spent gaming). The findings suggest that individuals with IGD who spend more time gaming may have more memories of previous gaming experiences when exposed to internet-gaming cues or may show a greater identification with the virtual world given that regions of the ECN have been implicated in theory of mind and self-other distinctions.

Ma et al. (2021) described less engagement of the left and right fronto-parietal networks during post-error slowing in individuals with IGD. Both the left and right fronto-parietal networks may be involved in different dimensions of cognitive control. Therefore, decreased activity in these networks may imply that individuals with IGD have difficulty using them to adjust response strategies to balance the opposing demands of stop-and-go trials and inhibit motor responses after errors.

Wang, Zhang et al. (2018) in their ICA study of 18 male individuals with IGD and 19 control participants identified differences between both groups with respect to functional networks, especially in the right ECN during an addiction-related Stroop task. Specifically, IGD individuals, compared to control participants, presented different functional connectivity in this network, with increased connectivity involving the middle temporal gyrus, superior temporal gyrus and middle frontal gyrus. This finding may suggest that individuals with IGD present abnormally enhanced cognitive-control processing towards gaming-related cues, perhaps to try to exert cognitive control over responses. In this vein, the ICA results by Wang et al. (2019), who explored ECN in 18 male individuals with IGD and 20 males with recreational internet game use, showed enhanced functional connectivity in the ECN in individuals with IGD, in comparison with the recreational-game-use group in probability and certain conditions. However, individuals with IGD had difficulty controlling their risky behaviours, tending to prefer lower probability and higher risky choices, possibly due to their higher levels of impulsivity. Similarly, by using ICA and a delay-discounting task, Wang et al. (2017) observed that individuals with IGD exhibited greater functional connectivity of the ECN, compared to control participants when selecting options related to now (versus later). Thus, individuals with IGD may have experienced greater demands on the behavioural inhibition system, demonstrating an additional cognitive effort to select among possible options. However, the increased functional connectivity of the ECN in these individuals could not inhibit their impulses, given that they typically exhibit difficulties in behavioural control, so they showed a greater tendency towards the new options. Yip et al. (2018) identified through ICA a decreased engagement of a fronto-cingulo-parietal network in individuals with IGD, in comparison to controls.

### Salience network

The SN mainly comprises the anterior insula, the dorsal anterior cingulate cortex (dACC) and the fronto-insular cortex (FIC). Moreover, the amygdala and striatum may contribute to this network (Menon and Uddin 2010; Borsook et al. 2013). The SN is involved in the detection of salient stimuli, both internal and external, to direct behaviour and maintain homeostasis (Toga 2015). The insula is responsible for detecting salient stimuli and initiating opportune control processing in response (Craig 2009; Menon and Uddin 2010). The SN also contributes to switching between the other two networks mentioned (DMN and ECN), thus facilitating orientation towards external versus internal stimuli (Sridharan et al. 2008; Figure 1).

Regarding IGD, it has been suggested that individuals with this disorder show reduced functional connectivity between the dACC and other brain areas within the SN (Chun et al. 2020). Furthermore, in IGD versus control participants, a positive association between reward sensitivity and effective connectivity in the ventral striatum of the SN has been observed (Chun et al. 2020). Other authors have highlighted the role of the SN in error processing, both in individuals with IGD and in control participants, given that both groups seem to activate the bilateral insula and the anterior cingulate cortex (Ko et al. 2014).

Some studies have explored the specific role of the SN in IGD using ICA. Lee et al. (2020) explored, using ICA, the SN, taking into account the bilateral anterior insular cortex. In a sample of 17 male adolescents with IGD and 18 control participants, the authors observed aberrant functional connectivity of the SN with the left pSTS only in adolescents with IGD. Furthermore, functional connectivity between the SN and pSTS was associated with proneness to internet addiction and self-reported cognitive problems. The pSTS is a relevant part of the social brain network. Therefore, excessive use of social stimuli linked to video games may alter the interaction between the SN and the social brain network (generating stronger functional connectivity between both networks), consequently possibly producing cognitive problems and executive alterations. In this vein, Wang et al. (2019) explored by ICA the anterior SN in 18 male individuals with IGD and 20 males with recreational internet game use. Differential connectivity of the anterior SN was identified in individuals with IGD, and this may be related to alterations in the regulation of risky behaviour and, consequently, to impaired cognitive control in IGD. Xing et al. (2014) observed a decreased fractional anisotropy in the SN, specifically in ACC/right-insula tracts, in individuals with IGD, in comparison with control participants. Yuan et al. (2016) found using ICA an interaction between the SN and right ECN (an influence of the ACC to rDLPFC) during resting state, reduced in the 28 individuals with IGD, in comparison with control participants. Finally, Zhang et al. (2017) explored the three networks together and found increased SN-DMN connectivity in individuals with IGD.

### Limitations and future research

The studies using ICA included in this narrative review have several limitations that should be considered. First, the sample sizes of several investigations were relatively small, which may weaken the rigour of the results. Therefore, studies with larger sample sizes are needed to draw stronger conclusions. Second, most studies include exclusively males with IGD. Although IGD appears to be a more prevalent disorder in males, further studies examining the neurobiology of IGD in females, as well as possible gender-related differences, are needed. Third, studies often included individuals with IGD recruited from educational centres, so the results may not generalise to broader populations. More studies focussed on treatment-seeking individuals with IGD are needed. Fourth, all studies included in the present narrative review have been conducted in Asia, mostly in China. Therefore, more studies using ICA to interrogate data from IGD participants from multiple other jurisdictions are needed to address possible cultural influences and other factors that may limit the generalisability of the findings that exist to date. Fifth, most studies have used the Internet addiction test (IAT) (Young 2009) to assess IGD, but it is not a specific psychometric tool for IGD. Similarly, most studies use the nine diagnostic criteria for IGD that were suggested in the DSM-5 (American Psychiatric Association 2013). Future studies should also use the diagnostic criteria accepted by ICD-11 (World Health Organization 2020). Sixth, the cross-sectional design is prevalent in included studies, so it is not possible to determine whether the alterations in brain connectivity observed in individuals with IGD are a consequence of the disorder or a predisposing factor. Therefore, longitudinal studies exploring these factors at different developmental stages of participating individuals are needed. Seventh, numerous included studies do not examine with precision the presence of psychiatric comorbidities, which could be influencing the described findings. Finally, task-based studies are subject to possible biases attributable to behavioural assessments, which should be taken into account when interpreting the results. Moreover, many of the tasks differed among studies, and there appears to be no uniformly agreed-upon approach for studying the neural correlates of IGD. Thus, while ICA studies have advanced the understanding of the neurobiology of IGD, additional research is needed.

## Conclusion

The DMN, ECN and SN have been explored using ICA in individuals with IGD. Most studies have identified alterations in these three brain networks in individuals with IGD, and these networks may be involved in the development and maintenance of IGD. However, more studies are needed in order to deepen an understanding of the specific roles of each in the symptomatology and treatment of IGD.
